# The relationship between sport motivation and problematic social networking sites use among adolescents: a study integrating mediation modeling and network analysis

**DOI:** 10.3389/fpsyg.2026.1920623

**Published:** 2026-09-23

**Authors:** Qian Zhao, Mingqin Geng, Huijuan Wang, Hailing Bai

**Affiliations:** 1 Department of Public Sports, Hebei North University, Zhangjiakou, Hebei, China; 2 Shijiazhuang College of Applied Technology, Shijiazhuang, Hebei, China; 3 Department of Physical Education, Xuanhua Vocational College of Science & Technology, Zhangjiakou, Hebei, China; 4 Hebei Jiaotong Vocational and Technical College, Shijiazhuang, Hebei, China

**Keywords:** adolescents, coping styles, mediation, network analysis, problematic social networking use, sport motivation

## Abstract

**Objective:**

This study aimed to examine the relationships between different types of sport motivation and problematic social networking use (PSNSU) among adolescents, to test the indirect associations through positive coping and negative coping, and to further investigate the conditional associations among specific psychological components using item-level network analysis.

**Methods:**

Data from 1,635 adolescents were analyzed. Sport motivation, coping styles, and PSNSU were assessed using the Sport Motivation Scale, the Simplified Coping Style Questionnaire, and the Problematic Social Networking Use Scale, respectively. Correlation analysis, parallel mediation analysis, and network analysis were conducted to examine the associations among autonomous motivation, controlled motivation, amotivation, positive coping, negative coping, and PSNSU.

**Results:**

Autonomous motivation was significantly and negatively associated with PSNSU, whereas controlled motivation and amotivation were both significantly and positively associated with PSNSU. Parallel mediation analyses showed significant indirect associations between all three types of sport motivation and PSNSU through positive coping and negative coping. The indirect association of autonomous motivation with PSNSU was more strongly reflected in the positive coping pathway, whereas those of controlled motivation and amotivation were more strongly reflected in the negative coping pathway. Network analysis further showed that positive coping was primarily connected with PSNSU through negative cross-community associations, whereas negative coping was primarily connected with PSNSU through positive cross-community associations. Several positive coping and PSNSU items also showed relatively high statistical importance within the overall network.

**Conclusion:**

Different types of sport motivation showed distinct patterns of association with adolescent PSNSU, while positive coping and negative coping exhibited indirect associations in opposite directions. Taken together, the construct-level parallel mediation analysis and item-level network analysis suggest that distinguishing different types of sport motivation and coping styles may provide a more nuanced understanding of the psychological characteristics associated with PSNSU among adolescents.

## 1 Background

As social networking has become increasingly integrated into adolescents' daily learning, social interactions, and recreational activities, problematic social networking use (PSNSU) has emerged as an important behavioral health concern. PSNSU is primarily characterized by difficulties in controlling social networking use, excessive engagement, and associated impairments in academic functioning, sleep, and offline social interactions (Elphinston and Noller, 2011; Hussain and Starcevic, 2020). In recent years, problematic social networking use has attracted increasing attention in the field of adolescent health worldwide. International evidence suggests that problematic patterns of social networking use among adolescents are not confined to any single country or cultural context. A 2022 survey conducted in the WHO European Region found that approximately 11% of nearly 2,80,000 adolescents across 44 countries and regions exhibited problematic social media use (Boniel-Nissim et al., 2024), indicating that this issue is relatively widespread among adolescents. Internet and social networking use are also highly prevalent among Chinese minors. In the context of academic pressure, interpersonal conflict, or negative emotional experiences, some adolescents may increasingly turn to social networking as a means of emotion regulation or psychological compensation, which may in turn be associated with higher levels of PSNSU. Therefore, identifying real-life psychological factors associated with PSNSU among adolescents is important for developing a more comprehensive understanding of this behavioral health concern.

### 1.1 The relationship between sport motivation and problematic social networking use

Sport motivation (SM) reflects why individuals participate in sport and the forms of regulation that drive their sport-related behavior (Teixeira et al., 2012). This concept differs from physical activity level, which mainly describes the frequency, intensity, or duration of an individual's actual participation in physical activity, whereas sport motivation focuses on the psychological reasons underlying sport participation and the quality of behavioral regulation. Therefore, even when two adolescents have similar levels of physical activity, their participation in sport may arise from different motivational sources, such as personal interest and perceived value, external demands and internal pressure, or a lack of clear intention to participate. According to Self-Determination Theory, different forms of sport motivation do not simply reflect higher or lower levels of motivation, but rather represent qualitatively different forms of behavioral regulation (Ryan and Deci, 2000). Autonomous motivation (AM) is primarily based on personal interest in and perceived value of sport, controlled motivation (CM) is more strongly driven by external demands or internal pressures, whereas amotivation (Amo) reflects a lack of clear intention to participate in sport and insufficient effective regulation. Therefore, compared with examining physical activity volume alone, distinguishing among different types of sport motivation can provide additional information about the psychological regulation underlying adolescents' sport participation and may help explain differential associations between motivational characteristics and PSNSU. Previous research has mainly examined the relationships between sport-related factors and problematic Internet-related behaviors using behavioral indicators such as physical activity level, exercise frequency, or sedentary behavior, with overall findings suggesting that higher levels of physical activity are associated with lower levels of problematic digital media use (Xiao et al., 2022; Zhao et al., 2024; Zhu et al., 2025). These studies indicate that actual participation in physical activity may be related to digital behaviors, but the amount of physical activity itself does not explain why individuals participate in sport or distinguish the quality of regulation underlying different forms of sport participation. Sport motivation therefore provides a psychological perspective that differs from the amount of sport-related behavior. Existing research has begun to suggest an association between sport motivation and problematic Internet-related behaviors (Wu et al., 2026), but whether different types of sport motivation show distinct patterns of association with PSNSU remains insufficiently studied. From the perspective of Self-Determination Theory, autonomous motivation reflects a higher degree of self-determined regulation and may be associated with lower levels of PSNSU (Ren et al., 2026; Shan et al., 2025); in contrast, controlled motivation relies more heavily on internal or external pressures, whereas amotivation reflects weaker behavioral regulation, and both may be associated with higher levels of PSNSU (Nurmi et al., 2016; Xiao et al., 2022). Based on the evidence above, this study proposed that autonomous motivation would be negatively associated with PSNSU, whereas controlled motivation and amotivation would be positively associated with PSNSU.

### 1.2 Indirect associations through coping styles

Beyond the direct associations between different types of sport motivation and PSNSU, coping styles may represent an important psychological link for understanding the relationship between the two. According to Self-Determination Theory, different qualities of sport motivation reflect different degrees of self-determined behavioral regulation. Autonomous motivation is primarily grounded in interest, value identification, and active choice, and may therefore be more closely associated with coping tendencies such as active problem solving, seeking support, and positive adjustment; in contrast, controlled motivation is more strongly driven by internal or external pressures, whereas amotivation reflects weaker behavioral regulation, and both may be more closely related to negative coping tendencies such as avoidance, withdrawal, or dependence. Furthermore, the transactional model of stress and coping proposes that, when individuals encounter external demands or challenging situations, they adopt corresponding cognitive and behavioral coping strategies based on their appraisal of the situation and available coping resources, and that different coping styles may be associated with different adaptive outcomes (Lazarus and Folkman, 1984). Therefore, the quality of behavioral regulation reflected in different types of sport motivation may be associated with different coping tendencies, while positive coping and negative coping may in turn be differentially associated with PSNSU. Coping styles (CS) refer to the cognitive and behavioral strategies individuals use to manage stress, regulate emotional responses, and adapt to external demands or adverse situations. According to the ways in which individuals respond to stress, CS are generally divided into positive coping (PC) and negative coping (NC) (Carver et al., 1989). Positive coping commonly includes problem solving, actively seeking help, rational appraisal, and emotion regulation. These strategies emphasize actively confronting stressors and making use of support resources available in the real world. In contrast, negative coping is commonly characterized by avoidance, denial, fantasy, self-blame, or withdrawal. Although such responses may temporarily reduce psychological discomfort, they are generally less effective in addressing the underlying problems (Xie, 1998). Sport motivation (SM) reflects the psychological reasons underlying adolescents' participation in sport as well as their level of self-regulation during physical activity. Adolescents with higher levels of autonomous motivation generally show stronger identification with the value of sport and a higher degree of self-determined regulation, and may therefore be more inclined to adopt positive coping strategies such as active adjustment and problem solving when facing stress (Smith et al., 2011; Zhang et al., 2025). In contrast, controlled motivation relies more heavily on pressure-driven regulation, whereas amotivation reflects weaker behavioral regulation, and both may be more likely to be associated with avoidance, withdrawal, or other forms of negative coping (Fuentealba-Urra et al., 2023; Dong et al., 2024). Previous research also provides some empirical support for these theoretical associations. Higher levels of self-determined sport motivation are generally associated with more positive behavioral regulation and coping tendencies, whereas weaker behavioral regulation is more likely to be associated with negative coping styles such as avoidance and withdrawal (Smith et al., 2011; Fuentealba-Urra et al., 2023; Dong et al., 2024; Zhang et al., 2025). At the same time, coping styles are also closely associated with PSNSU and other problematic Internet-related behaviors. Existing studies have shown that positive coping styles, such as problem solving and seeking support, are generally associated with lower levels of problematic Internet use, whereas avoidance, withdrawal, and passive coping are more frequently associated with higher levels of problematic use (Chwaszcz et al., 2018; McNicol and Thorsteinsson, 2017; Ma et al., 2022; Dong et al., 2024). This pattern is consistent with the transactional model of stress and coping, which suggests that different coping styles may correspond to different adaptive outcomes. Therefore, different types of sport motivation may show distinct indirect associations with PSNSU through positive coping and negative coping. Specifically, autonomous motivation may be more closely associated with positive coping, whereas controlled motivation and amotivation may be more closely associated with negative coping, thereby showing indirect associations with PSNSU in different directions (McNicol and Thorsteinsson, 2017; Varela et al., 2023). Based on the evidence above, this study proposed H2: autonomous motivation would show a negative indirect association with PSNSU through positive coping and negative coping, whereas controlled motivation and amotivation would show positive indirect associations with PSNSU through positive coping and negative coping.

### 1.3 Cross-sectional network analysis

To further clarify the complex patterns of association among sport motivation, coping styles, and problematic social networking use, the present study incorporated cross-sectional network analysis in addition to traditional mediation analysis. Within the network framework, specific items reflecting SM, positive coping, negative coping, and PSNSU were treated as nodes, while edges between nodes represented conditional associations among psychological and behavioral components after controlling for all other nodes in the network. Compared with traditional approaches that primarily examine relationships at the scale-score level, network analysis provides a more fine-grained item-level perspective. It can reveal connections both within and between constructs and identify key nodes that occupy central positions or serve as bridges within the overall network structure (Epskamp et al., 2018; Jones et al., 2021). The use of cross-sectional network analysis enabled this study to examine the co-occurrence patterns of SM, CS, and PSNSU at the same time point. More importantly, it allowed comparison of the different roles played by positive coping and negative coping within the network. Positive coping may be associated with lower levels of PSNSU through problem solving, emotional expression, support seeking, and positive cognitive appraisal (Lu et al., 2022; Wegmann et al., 2023). In contrast, negative coping may be associated with a higher risk of PSNSU through avoidance, passive waiting, dependence on others, and self-comforting strategies (Chwaszcz et al., 2018). By identifying the connections among these specific items, the present study can more directly indicate which CS nodes may show protective or risk-related bridging associations between SM and PSNSU (Castro et al., 2019). Therefore, cross-sectional network analysis complements traditional mediation models, which mainly describe overall pathway effects but provide limited information about the roles of specific psychological components. In recent years, network analysis has been increasingly applied in research on mental health, addictive behaviors, and adolescent development. Its main advantage lies in its ability to characterize conditional associations within complex psychological systems and to identify candidate nodes that warrant further examination in future longitudinal or experimental research. By integrating mediation analysis and network analysis, the present study not only examined whether indirect associations between SM and PSNSU existed through CS, but also explored which specific psychological and behavioral components may deserve greater attention in future prevention and intervention efforts.

### 1.4 The present study

Overall, existing research has focused primarily on the relationships between physical activity level, exercise frequency, or sedentary behavior and problematic Internet-related behaviors, largely addressing whether “how much adolescents exercise” is associated with their digital behavior (Xiao et al., 2022; Zhao et al., 2024; Zhu et al., 2025). However, comparatively limited attention has been paid to “why adolescents participate in sport” and whether different qualities of sport-related regulation are differentially associated with PSNSU. Physical activity mainly reflects the level of actual behavior, whereas sport motivation reflects the psychological reasons underlying sport participation and the quality of behavioral regulation; therefore, the two represent indicators at different levels. Distinguishing autonomous motivation, controlled motivation, and amotivation can complement behavioral measures of sport participation by providing a psychological-regulation perspective on factors associated with adolescent PSNSU. Furthermore, evidence remains limited regarding how different types of sport motivation may be linked to PSNSU through more proximal psychological processes. Coping styles may represent one important psychological link. According to Self-Determination Theory, the higher degree of self-determined regulation reflected in autonomous motivation may be more closely associated with positive coping, whereas controlled motivation and amotivation may be more closely associated with negative coping; in turn, positive coping and negative coping may be associated with lower and higher levels of PSNSU, respectively.

Therefore, at the construct level, the present study developed three parallel mediation models, with AM, CM, and Amo separately specified as independent variables, PC and NC as parallel mediators, and PSNSU as the dependent variable, in order to compare the patterns of indirect associations between different types of sport motivation and PSNSU through the two coping styles (see Figure 1). Building on this analysis, an item-level network analysis was further conducted, in which AM, CM, Amo, PC, NC, and PSNSU were divided into six theoretically defined communities to examine conditional associations among specific items and identify nodes characterized by relatively high node strength and cross-community connectivity. Through this analytical progression from the construct level to the item level, the present study not only examined the overall patterns of association among different types of sport motivation, coping styles, and PSNSU, but also further revealed more fine-grained item-level connections within and between these constructs, thereby providing more specific evidence for understanding the relationship between the quality of psychological regulation underlying sport participation and adolescent PSNSU.

**Figure 1:**
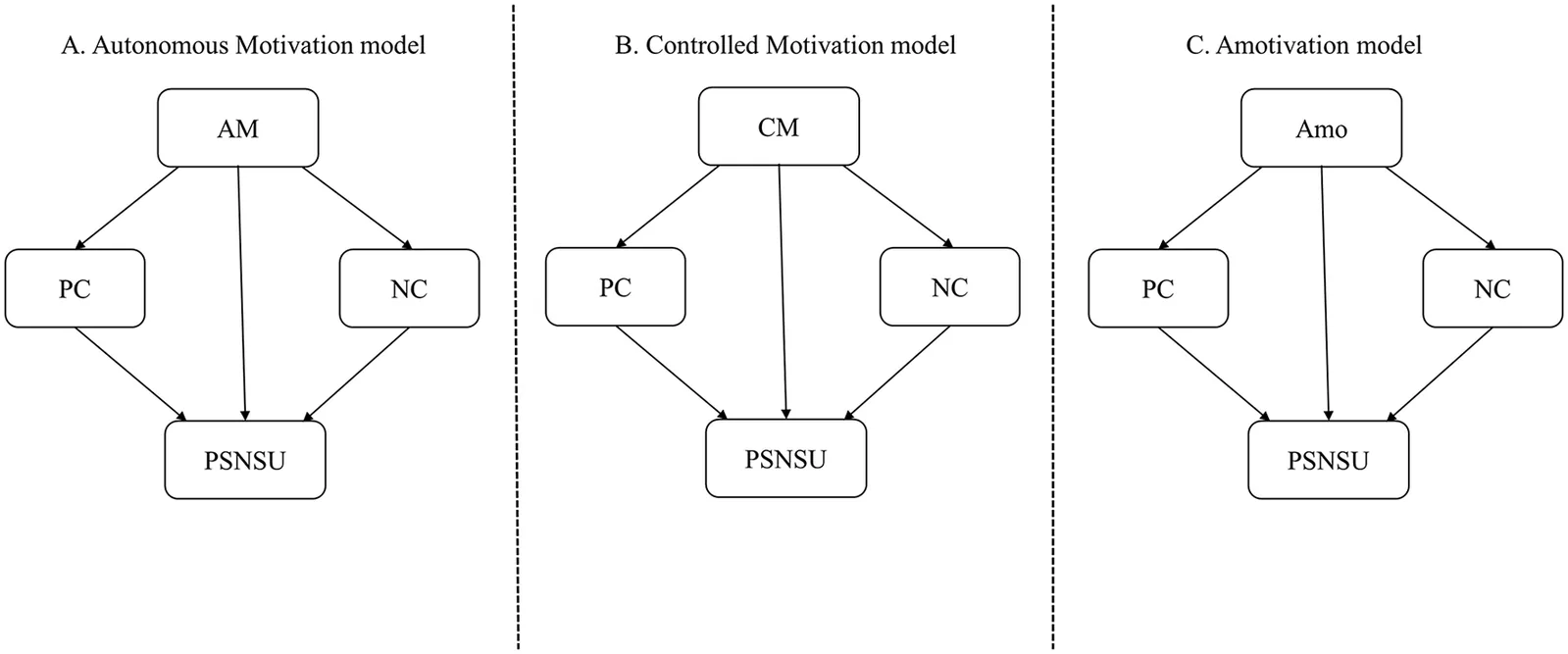
Parallel mediation models of positive and negative coping in the associations between sport motivation dimensions and problematic social networking use. **(A)** Autonomous motivation model; **(B)** controlled motivation model; **(C)** amotivation model.

## 2 Methods

### 2.1 Participants

This study employed non-probability convenience sampling. Five middle schools in Sichuan Province, China, were selected based on school accessibility, willingness to cooperate, and the feasibility of questionnaire administration. After obtaining permission from the schools, teachers assisted in introducing the study to eligible students and distributing the questionnaire link or QR code. Students independently completed the anonymous online questionnaire on an informed and voluntary basis, and teachers were not involved in the students' specific responses. Questionnaires were collected anonymously through the Wenjuanxing platform, and no directly identifying information, such as students' names or student identification numbers, was recorded. To prevent duplicate submissions, the platform was configured to allow only one submission per device. A total of 2,106 questionnaires were collected. Data collection was conducted from September to November 2025. To reduce potential common method bias at the source, several procedural controls were implemented during questionnaire administration. First, items from different dimensions were presented on separate pages. Second, a mandatory 15-second interval was imposed before participants could proceed to the next page, with the aim of reducing response inertia resulting from continuous scale completion. These procedures were intended to improve response quality and minimize the likelihood of mechanical or repetitive responding. Invalid questionnaires were excluded according to predefined data-quality criteria to reduce the influence of careless or random responses. The exclusion criteria were as follows: (1) based on methodological recommendations that an average response time of less than 2 s per substantive item may indicate excessively rapid responding (Huang et al., 2012; Curran, 2016), questionnaires with a total completion time of less than 112 s were classified as rapid responses and excluded; (2) the longest string of identical responses (LongString) index was used to identify mechanically repetitive responding (Huang et al., 2012; Curran, 2016). For the 28-item Sport Motivation Scale and the 20-item coping styles scale, selecting the same response option for 15 or more consecutive items and 11 or more consecutive items, respectively, was considered abnormal consecutive responding. Because the PSNSU scale contains only eight items and its item content is relatively concentrated, questionnaires were not classified as invalid solely on the basis of the LongString results for this scale; and (3) questionnaires with clearly abnormal key information or evidence of random or invalid responding were excluded. After screening, 132 questionnaires were excluded because of rapid responding, 215 because of abnormal consecutive responding, and 124 because of missing key information. The final analytical sample consisted of 1,635 valid questionnaires. Participants ranged in age from 12 to 19 years, with a mean age of 15.00 years (SD = 1.79). Of the participants, 848 were boys and 787 were girls, accounting for 51.87% and 48.13% of the sample, respectively. Regarding grade distribution, 350 participants were in Grade 7 (21.41%), 372 in Grade 8 (22.75%), 289 in Grade 9 (17.68%), 244 in Grade 10 (14.92%), 196 in Grade 11 (11.99%), and 184 in Grade 12 (11.25%). In addition, 333 participants were left-behind children (20.37%) and 1,302 were non-left-behind children (79.63%); 592 were boarding students (36.21%) and 1,043 were non-boarding students (63.79%); and 748 were only children (45.75%) and 887 were non-only children (54.25%). With regard to place of residence, 782 participants lived in urban areas (47.83%) and 853 lived in rural areas (52.17%). Prior to data collection, the study was conducted in accordance with the Declaration of Helsinki and relevant ethical guidelines for research involving human participants. Participation was entirely voluntary. Before questionnaire completion, students and their guardians were informed of the study purpose, survey content, anonymity, data confidentiality, and their right to withdraw from the study at any time. Written informed consent was obtained from both the students and their parents or legal guardians before data collection. The study protocol was reviewed and approved by the Academic Ethics Committee of Hebei North University (Approval No. 2026XY009).

### 2.2 Measures

#### 2.2.1 Sport motivation

Sport motivation was assessed using the Sport Motivation Scale (SMS) developed by Pelletier et al. (1995) and the Chinese version translated by Peng (2011), which has been widely used in China and has demonstrated good reliability and validity (Li, 2025). The scale consists of 28 items rated on a 7-point Likert scale ranging from 1 (“does not correspond at all”) to 7 (“corresponds exactly”), with no reverse-scored items. The original scale includes seven forms of motivational regulation: intrinsic motivation to know, intrinsic motivation toward accomplishment, intrinsic motivation to experience stimulation, identified regulation, introjected regulation, external regulation, and amotivation. According to Self-Determination Theory, different forms of sport motivation regulation can be further grouped into higher-order motivational categories according to the degree of autonomy in behavioral regulation (Ryan and Deci, 2000). In the present study, the three forms of intrinsic motivation and identified regulation were grouped as autonomous motivation, introjected regulation and external regulation were grouped as controlled motivation, and amotivation was retained as an independent construct. This classification is consistent with previous sport and adolescent research using the original SMS (Jaakkola et al., 2008; Rottensteiner et al., 2015). Higher scores on autonomous motivation indicate greater autonomy in sport participation; higher scores on controlled motivation indicate that sport participation is more strongly driven by external or internal pressures; and higher scores on amotivation indicate a weaker intention to participate in sport. In the present study, autonomous motivation demonstrated good internal consistency, with a Cronbach's α of 0.842 and a McDonald's ω of 0.848; controlled motivation had a Cronbach's α of 0.781 and a McDonald's ω of 0.789; and amotivation had a Cronbach's α of 0.746 and a McDonald's ω of 0.758. Confirmatory factor analysis was further conducted to examine the measurement structure of the three motivational constructs, and the results indicated good model fit: χ^2^(347) = 1,016.40, χ^2^/df = 2.929, NFI = 0.923, CFI = 0.948, TLI = 0.939, IFI = 0.949, RMSEA = 0.033, and SRMR = 0.041, indicating good overall model fit.

#### 2.2.2 Coping styles

Coping styles were assessed using the simplified coping style questionnaire (SCSQ) developed by Xie (1998). The questionnaire consists of 20 items rated on a 4-point Likert scale and is designed to assess individuals' coping tendencies when facing stress, difficulties, or adverse situations. The questionnaire includes two dimensions: positive coping and negative coping, with Items 1–12 assessing positive coping and Items 13–20 assessing negative coping. In the present study, the internal consistency of the two dimensions was examined separately. For positive coping, Cronbach's α was 0.729 and McDonald's ω was 0.732; for negative coping, Cronbach's α was 0.82 and McDonald's ω was 0.86. A correlated two-factor confirmatory factor analysis model was further specified according to the theoretical structure of the original questionnaire, with the two latent factors allowed to correlate. The model fit indices were χ^2^/df = 3.378, NFI = 0.882, CFI = 0.914, TLI = 0.903, IFI = 0.914, RMSEA = 0.037, and SRMR = 0.036, indicating an overall good fit of the two-factor model. Higher scores on positive coping indicate a greater tendency to adopt positive coping strategies, whereas higher scores on negative coping indicate a greater tendency to adopt negative coping strategies.

#### 2.2.3 Problematic social networking use

Problematic social networking use was assessed using the Problematic Social Networking Use Scale revised by Wei (2018), which was adapted from the Facebook Intrusion Questionnaire developed by Elphinston and Noller (2011). The scale consists of 8 items and is primarily used to assess problematic consequences associated with excessive use of social networking sites, including interference with academic activities, impaired sleep, disruption of offline social interactions, withdrawal-like responses, and difficulties in controlling use. The scale is typically scored as a unidimensional measure. In the present study, a 4-point response format was used in accordance with the questionnaire design, ranging from 1 = “never” to 4 = “almost every day.” All items were scored in the same direction, with no reverse-scored items. Higher total scores indicate higher levels of PSNSU. In the present study, the scale demonstrated good internal consistency, with a Cronbach's α of 0.800 and a McDonald's ω of 0.801, and the KMO value was 0.888. Confirmatory factor analysis indicated good model fit: χ^2^/df = 4.411, RMSEA = 0.044, SRMR = 0.030, GFI = 0.997, NFI = 0.971, CFI = 0.977, and IFI = 0.977.

### 2.3 Data analysis

SPSS 27.0 was used for descriptive statistics, Pearson correlation analysis, internal consistency reliability testing, common method bias testing, and structural validity analysis of the measurement instruments. Parallel indirect effects were estimated using Model 4 of the PROCESS 4.2 macro. Based on the classification of different forms of sport motivation regulation derived from Self-Determination Theory, three parallel mediation models were constructed separately, with autonomous motivation, controlled motivation, and amotivation specified as the independent variables, problematic social networking use as the dependent variable, and positive coping and negative coping as parallel mediators. Sex and age were included as covariates in all models. Because participants were recruited from five schools, school-level clustering effects were further examined. The results showed that the school-level ICCs for autonomous motivation (ICC <0.001), controlled motivation (ICC = 0.009), amotivation (ICC <0.001), positive coping (ICC = 0.002), negative coping (ICC = 0.005), and PSNSU (ICC <0.001) were all below 0.01, indicating no substantial school-level clustering effect. Bias-corrected Bootstrap procedures with 5,000 resamples were used to estimate the specific indirect effects, total indirect effects, and their corresponding 95% confidence intervals. An indirect effect was considered statistically significant when the 95% Bootstrap confidence interval did not include zero.

In addition, item-level network analysis was conducted in R using the qgraph, bootnet, networktools, psych, openxlsx, and svglite packages. Because all scale items in the present study were ordinal Likert-type variables, polychoric correlations were used for network estimation. Specifically, cor_auto was used to automatically identify ordinal variables and generate the corresponding correlation matrix, after which the EBICglasso method was applied to estimate a regularized partial correlation network, with the EBIC hyperparameter γ set to 0.50. To ensure consistency between the network community structure, Self-Determination Theory, and the preceding construct-level analyses, the 56 nodes were divided into six theoretically defined communities: autonomous motivation (AM, 16 items), controlled motivation (CM, 8 items), amotivation (Amo, 4 items), positive coping (PC, 12 items), negative coping (NC, 8 items), and problematic social networking use (PSNSU, 8 items). Specifically, the three intrinsic motivation dimensions and identified regulation items from the original SMS were assigned to the AM community, introjected regulation and external regulation items were assigned to the CM community, and the amotivation items constituted the Amo community. Node strength, bridge strength, and bridge expected influence were then calculated to identify important nodes within the overall network and nodes showing strong cross-community connections among the six theoretical communities. Network edge-weight accuracy was evaluated using 1,000 nonparametric Bootstrap samples, and the stability of node strength centrality was assessed using 1,000 case-dropping Bootstrap samples. Centrality stability was evaluated using the correlation stability coefficient (CS-coefficient).

## 3 Results

### 3.1 Common method bias

Potential common method bias was examined using Harman's single-factor test (Podsakoff et al., 2003). The results showed that five factors had eigenvalues greater than 1. The first unrotated factor accounted for 11.815% of the total variance, which was well below the recommended threshold of 40%. Thus, the results did not indicate that a single factor accounted for a substantial proportion of the variance.

### 3.2 Descriptive statistics and correlation analysis

The results of the Pearson correlation analysis are presented in Table 1. Autonomous motivation (AM) was significantly and positively correlated with positive coping (PC; *r* = 0.315, *p* < 0.001), and significantly and negatively correlated with both negative coping (NC) and problematic social networking use (PSNSU; *r* = −0.185 to −0.250, all *p* < 0.001). In contrast, controlled motivation (CM) and amotivation (Amo) were both significantly and negatively correlated with PC, but significantly and positively correlated with NC and PSNSU. Among these associations, amotivation showed a relatively stronger correlation with NC (*r* = 0.335, *p* < 0.001). In addition, PC was significantly and negatively correlated with PSNSU (*r* = −0.350, *p* < 0.001), whereas NC was significantly and positively correlated with PSNSU (*r* = 0.380, *p* < 0.001). Overall, the direction of the correlations was largely consistent with the study hypotheses and provided preliminary support for further examining the parallel indirect associations through PC and NC.

**Table 1:** Descriptive statistics and correlations among the main study variables.

Variable	M	SD	1	2	3	4	5	6
1. AM	73.34	9.61	1					
2. CM	27.13	5.79	−0.568^***^	1				
3. Amo	13.95	3.47	−0.444^***^	0.367^***^	1			
4. PC	28.75	5.63	0.315^***^	−0.125^**^	−0.083^***^	1		
5. NC	20.43	4.21	−0.185^***^	0.275^***^	0.335^***^	−0.500^***^	1	
6. PSNSU	18.32	5.15	−0.250^**^	0.198^***^	0.194^**^	−0.350^**^	0.380^***^	1

AM, autonomous motivation; CM, controlled motivation; Amo, amotivation; PC, positive coping; NC, negative coping; PSNSU, problematic social networking use.

^**^p < 0.01.

^***^p < 0.001.

### 3.3 Indirect effect analysis

Parallel mediation models were further used to examine the indirect effects of different dimensions of sport motivation on PSNSU through PC and NC, and the results are presented in Table 2 and Figure 2. Path coefficients and direct effects are reported as standardized coefficients (β). For AM, AM was significantly and positively associated with PC (β = 0.320, *p* < 0.001) and significantly and negatively associated with NC (β = −0.180, *p* < 0.001); PC was significantly and negatively associated with PSNSU (β = −0.240, *p* < 0.001), whereas NC was significantly and positively associated with PSNSU (β = 0.190, *p* < 0.001). The direct association between AM and PSNSU remained significant (β = −0.140, *p* < 0.001). Bootstrap analysis showed that the indirect effect through PC was −0.077, 95% CI [−0.095, −0.060], the indirect effect through NC was −0.034, 95% CI [−0.049, −0.021], and the total indirect effect was −0.111, 95% CI [−0.132, −0.091], indicating significant indirect associations between AM and PSNSU through both PC and NC.

**Table 2:** Parallel mediation effects of positive and negative coping in the associations between sport motivation dimensions and problematic social networking use.

Model	X → PC (a_1_)	X → NC (a_2_)	PC → PSNSU (b_1_)	NC → PSNSU (b_2_)	Direct effect (c^′^)	Indirect via PC (a_1_b_1_)	Indirect via NC (a_2_b_2_)	Total indirect effect	Total effect (c)
Model 1 (AM)	0.320^***^	−0.180^***^	−0.240^***^	0.190^***^	−0.140^***^	−0.077 [−0.095, −0.060]	−0.034 [−0.049, −0.021]	−0.111 [−0.132, −0.091]	−0.251^**^
Model 2 (CM)	−0.120^**^	0.280^***^	−0.203^***^	0.240^**^	0.105^**^	0.024 [0.011, 0.039]	0.067 [0.049, 0.087]	0.091 [0.071, 0.113]	0.196^***^
Model 3 (Amo)	−0.080^*^	0.340^***^	−0.180^***^	0.270^***^	0.089^**^	0.014 [0.004, 0.027]	0.092 [0.071, 0.115]	0.106 [0.083, 0.130]	0.195^**^

AM, autonomous motivation; CM, controlled motivation; Amo, amotivation; PC, positive coping; NC, negative coping; PSNSU, problematic social networking use.

Values in brackets represent 95% bootstrap confidence intervals.

^*^p < 0.05.

^**^p < 0.01.

^***^p < 0.001.

**Figure 2:**
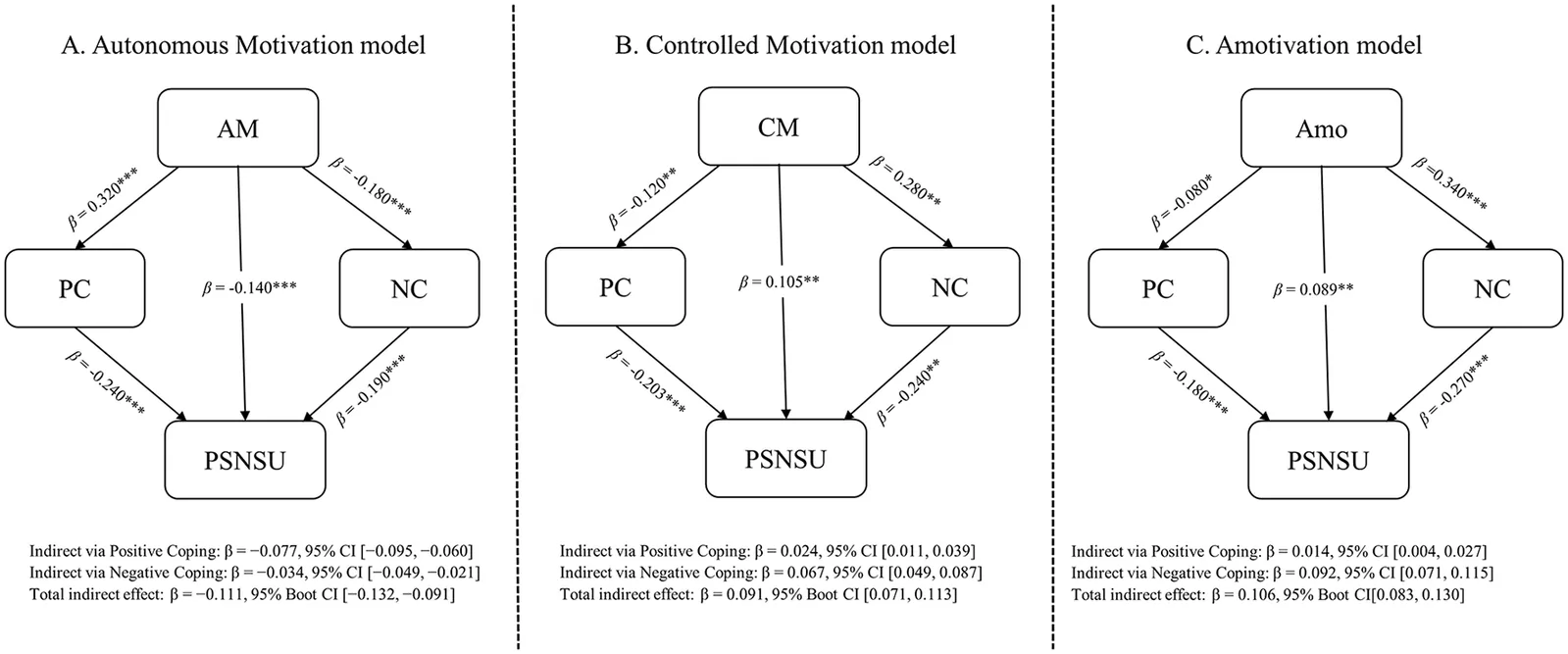
Parallel mediation models of positive and negative coping in the associations between sport motivation dimensions and problematic social networking use. **(A)** Autonomous motivation model; **(B)** controlled motivation model; **(C)** amotivation model.

For CM, CM was significantly and negatively associated with PC (β = −0.120, *p* < 0.01) and significantly and positively associated with NC (β = 0.280, *p* < 0.001). PC was significantly and negatively associated with PSNSU (β = −0.203, *p* < 0.001), whereas NC was significantly and positively associated with PSNSU (β = 0.240, *p* < 0.01). The direct association between CM and PSNSU remained significant (β = 0.105, *p* < 0.01). The indirect effect through PC was 0.024, 95% CI [0.011, 0.039], the indirect effect through NC was 0.067, 95% CI [0.049, 0.087], and the total indirect effect was 0.091, 95% CI [0.071, 0.113]. Neither Bootstrap confidence interval included zero, indicating significant indirect associations between CM and PSNSU through both PC and NC, with the indirect effect through NC being relatively larger.

For Amo, Amo was significantly and negatively associated with PC (β = −0.080, *p* < 0.05) and significantly and positively associated with NC (β = 0.340, *p* < 0.001); PC was significantly and negatively associated with PSNSU (β = −0.180, *p* < 0.001), whereas NC was significantly and positively associated with PSNSU (β = 0.270, *p* < 0.001). The direct association between Amo and PSNSU also remained significant (β = 0.089, *p* < 0.01). Bootstrap analysis showed that the indirect effect through PC was 0.014, 95% CI [0.004, 0.027], the indirect effect through NC was 0.092, 95% CI [0.071, 0.115], and the total indirect effect was 0.106, 95% CI [0.083, 0.130]. The indirect effect through NC was substantially larger than that through PC, indicating a relatively stronger indirect association between amotivation and PSNSU through NC.

Overall, significant indirect associations between all three types of sport motivation and PSNSU were observed through both positive coping and negative coping, but the patterns differed across motivation types: the indirect association of autonomous motivation was more strongly reflected in the positive coping pathway, whereas those of controlled motivation and amotivation were more strongly reflected in the negative coping pathway.

### 3.4 Network analysis among variables

#### 3.4.1 Overview of the item-level network analysis

The items assessing sport motivation, coping styles, and PSNSU were treated as network nodes in the item-level network analysis. Specifically, SM1–SM28 represented the sport motivation items, CS1–CS20 represented the coping styles items, and IA1–IA8 were the item codes used in the original dataset, corresponding to the eight items of the PSNSU scale. Based on Self-Determination Theory and the theoretical structure of each scale, the 56 nodes were further divided into six communities: autonomous motivation (AM, 16 items), controlled motivation (CM, 8 items), amotivation (Amo, 4 items), positive coping (PC, 12 items), negative coping (NC, 8 items), and PSNSU (8 items). Specifically, the three intrinsic motivation dimensions and identified regulation items from the SMS were assigned to the AM community, introjected regulation and external regulation items were assigned to the CM community, and the amotivation items constituted the Amo community; CS1–CS12 were assigned to the PC community, whereas CS13–CS20 were assigned to the NC community. For ease of interpretation, the scale, theoretical dimension, and brief description corresponding to each node are presented in Table 2. The network analysis included examination of the overall network structure, node strength centrality, bridge centrality, and network accuracy and stability. In the network graph, nodes from the six communities were distinguished using different colors. Edges between nodes represented conditional associations between two items after controlling for all other nodes in the network, with thicker edges indicating stronger associations. Centrality and bridge centrality indices were presented in standardized form to facilitate comparison of the relative importance of nodes within the overall network and across different communities.

#### 3.4.2 Network structure estimation

The network structure showed relatively clear item-level connection patterns among the six communities of AM, CM, Amo, PC, NC, and PSNSU. Overall, nodes within the same theoretical community were primarily positively connected, whereas cross-community associations between theoretically distinct communities showed different directions. Within the PSNSU community, all item-level connections were positive, with relatively strong edges observed for IA5–IA6, IA1–IA3, IA7–IA8, IA5–IA8, IA6–IA7, IA3–IA4, and IA1–IA2, indicating relatively close conditional associations among different PSNSU symptoms.

With regard to coping styles, connections within the positive coping community were predominantly positive, with relatively strong edges for CS2–CS3, CS7–CS9, CS8–CS9, CS2–CS4, CS8–CS10, and CS6–CS7. Connections within the negative coping community were likewise primarily positive, with prominent edges for CS14–CS15, CS17–CS19, CS17–CS20, CS16–CS20, CS13–CS15, and CS18–CS20. In contrast, all retained connections between positive coping and negative coping were negative, with CS12–CS13, CS11–CS15, CS12–CS15, and CS11–CS14 representing the stronger negative edges. These findings further indicate that the two coping styles showed distinct item-level association patterns.

For the connections between coping styles and PSNSU, all retained cross-community connections between positive coping and PSNSU were negative, with relatively strong negative edges for CS2–IA5, CS3–IA3, CS4–IA4, CS6–IA6, and CS2–IA7. In contrast, all retained connections between negative coping and PSNSU were positive, with relatively prominent positive edges for CS13–IA2, CS20–IA4, CS17–IA8, CS15–IA1, and CS18–IA1. Overall, positive coping items were mainly associated with lower levels of PSNSU symptoms, whereas negative coping items were mainly associated with higher levels of PSNSU symptoms.

Different sport motivation communities also showed relatively clear directional differences. All retained connections within autonomous motivation were positive, with relatively strong edges for SM27–SM25, SM20–SM18, SM2–SM7, and SM18–SM17; connections within controlled motivation were also predominantly positive, with SM21–SM16, SM9–SM14, and SM14–SM10 being particularly prominent. All retained connections between autonomous motivation and controlled motivation were negative, with relatively strong edges for SM13–SM14, SM25–SM22, SM8–SM14, and SM24–SM26; the connections between autonomous motivation and amotivation were also all negative. In contrast, the retained connections between controlled motivation and amotivation were mainly positive. All item-level connections within the amotivation community were shrunk to zero after regularization.

Among the cross-community connections between sport motivation, coping styles, and PSNSU, relatively few direct item-level connections were retained. Two positive connections, SM11–CS9 and SM1–CS7, were retained between autonomous motivation and positive coping, whereas no direct connections were retained between autonomous motivation and negative coping. All retained connections between autonomous motivation and PSNSU were negative, with SM18–IA2 and SM18–IA5 being relatively strong. Two negative connections, SM14–CS11 and SM14–CS9, were retained between controlled motivation and positive coping, whereas all direct item-level connections between controlled motivation and negative coping were shrunk to zero after regularization. All retained connections between controlled motivation and PSNSU were positive, with SM6–IA7 being relatively strong. No direct item-level connections were retained between amotivation and either positive coping or negative coping, whereas only one positive connection, SM5–IA5, was retained between amotivation and PSNSU.

Overall, the six-community network showed item-level patterns that were broadly consistent with the theoretical distinctions: positive coping and PSNSU were primarily connected through negative cross-community associations, whereas negative coping and PSNSU were primarily connected through positive cross-community associations; autonomous motivation was mainly negatively connected with controlled motivation and amotivation, whereas controlled motivation was mainly positively connected with amotivation. At the same time, some direct item-level connections between constructs were shrunk to zero after controlling for all other nodes in the network, indicating that construct-level overall associations do not necessarily correspond to a large number of direct item-level connections. Further distinguishing sport motivation into autonomous motivation, controlled motivation, and amotivation, and coping styles into positive coping and negative coping, helps provide a more fine-grained understanding of the local conditional associations among different theoretical dimensions. The network structure is presented in Figures 3a, b.

**Figure 3:**
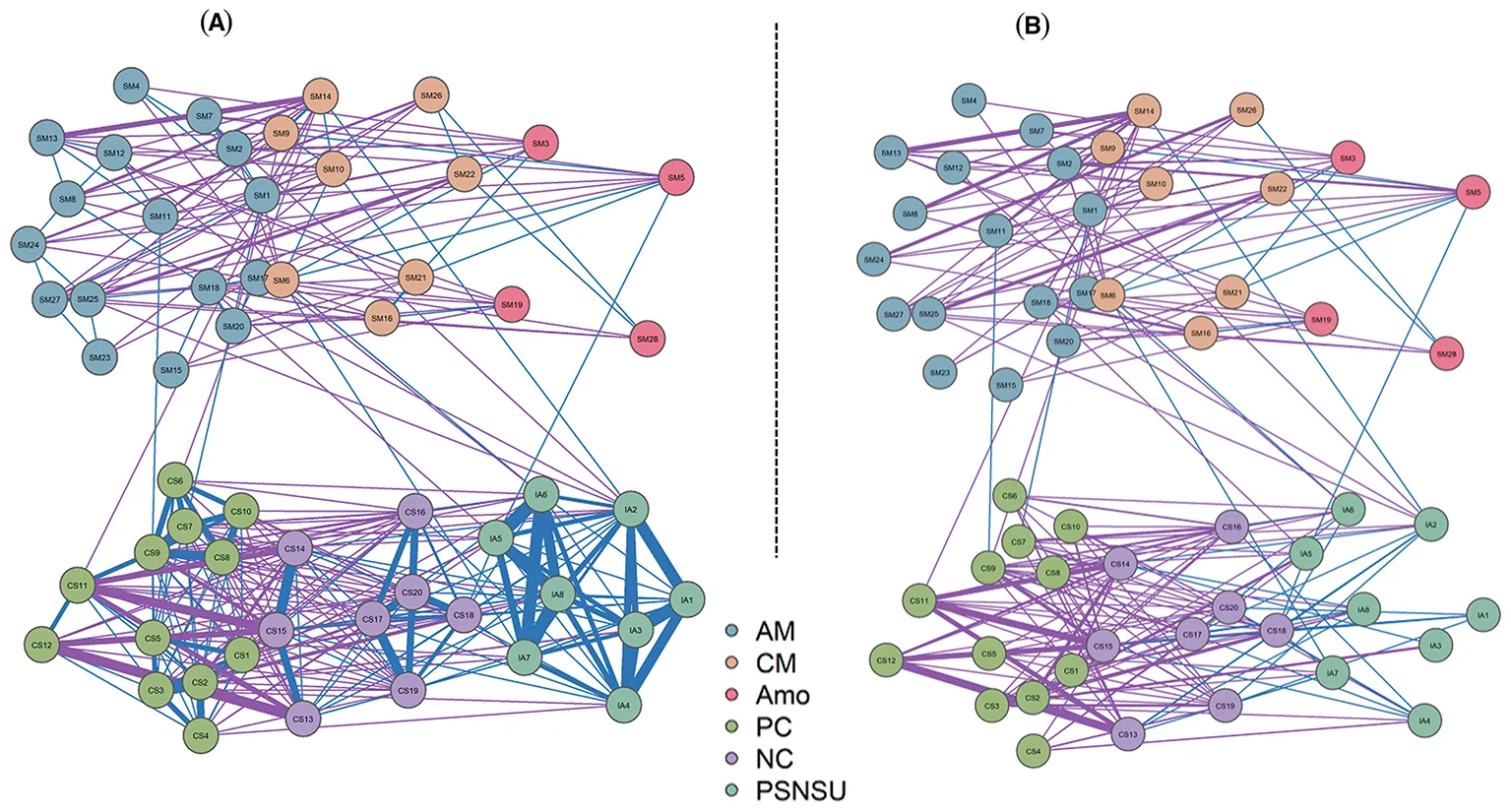
Network structure among the study variables. **(A)** and **(B)** show two visualizations of the estimated item-level network. Nodes represent individual items from each scale, and edges represent regularized partial correlations between nodes. Blue edges indicate positive connections, whereas purple edges indicate negative connections. SM, sport motivation; CS1–CS12, positive coping; CS13–CS20, negative coping; IA1–IA8, items of problematic social networking use.

#### 3.4.3 Node and bridge centrality analysis

Node strength analysis showed that CS9 had the highest node strength, followed by IA8, IA2, IA4, CS3, CS2, CS15, IA5, IA6, and IA7, indicating that these nodes had relatively high importance within the overall network. Among them, CS9 (“change previous ways of doing things or one's own problems”), CS2 (“talk with others and express inner concerns”), and CS3 (“try to see the positive side of things”) all belonged to the positive coping community, suggesting that active adjustment, emotional expression, and positive cognitive appraisal were relatively important psychological components in the overall network. CS15 belonged to the negative coping community and also showed relatively high node strength. Within the PSNSU community, IA8, IA2, IA4, IA5, IA6, and IA7 also showed relatively high node strength, indicating that symptoms related to impaired control, urges to use, disruption of real-life functioning, social feedback, emotion-related use, and withdrawal-like responses occupied relatively important positions in the overall network.

Bridge strength analysis showed that CS15 had the highest bridge strength, followed by CS13, CS11, SM14, CS14, CS19, CS9, CS12, CS16, and CS2. Compared with the three-community model, after autonomous motivation, controlled motivation, amotivation, positive coping, negative coping, and PSNSU were divided into six separate communities, nodes with relatively high bridge strength were mainly concentrated in the positive coping and negative coping communities, together with several sport motivation nodes. This indicates that these nodes had more numerous or stronger conditional connections with other theoretical communities. It should be noted that, within the six-community framework, bridge strength reflects the total strength of a node's connections with all communities outside its own community. Therefore, higher bridge strength does not mean that a node is primarily connected only with PSNSU, but rather reflects its overall degree of cross-community connectivity within the six-community network.

Bridge expected influence further reflected the overall direction of cross-community connections. The results showed that IA1, IA8, IA7, IA2, and IA4 exhibited relatively high positive bridge expected influence, indicating that the net cross-community associations of these PSNSU nodes with other communities were generally positive. In contrast, CS11, SM14, CS15, CS13, CS12, CS14, CS9, CS2, CS16, and CS19 showed relatively strong negative bridge expected influence, indicating that the net cross-community associations of these nodes with other communities were generally negative. Because the current bridge indices simultaneously consider all cross-community connections among the six communities of AM, CM, Amo, PC, NC, and PSNSU, the positive or negative direction of bridge expected influence should not be interpreted simply as indicating risk or protective effects, nor should it be interpreted solely according to whether a node belongs to the positive coping or negative coping dimension. Rather, it should be understood as reflecting the overall direction of a node's cross-community conditional associations.

Overall, the node strength results indicated that the relatively important nodes in the overall network were mainly concentrated in the positive coping, negative coping, and PSNSU communities. The bridge analysis further showed that nodes such as CS15, CS13, CS11, SM14, CS14, and CS19 exhibited relatively high levels of cross-community connectivity across the six communities. The six-community classification allowed the network analysis to further distinguish cross-community connections among different dimensions of sport motivation and coping styles, thereby complementing the construct-level findings from an item-level perspective. The results for node strength, bridge strength, and bridge expected influence are presented in Figures 4a–c.

**Figure 4:**
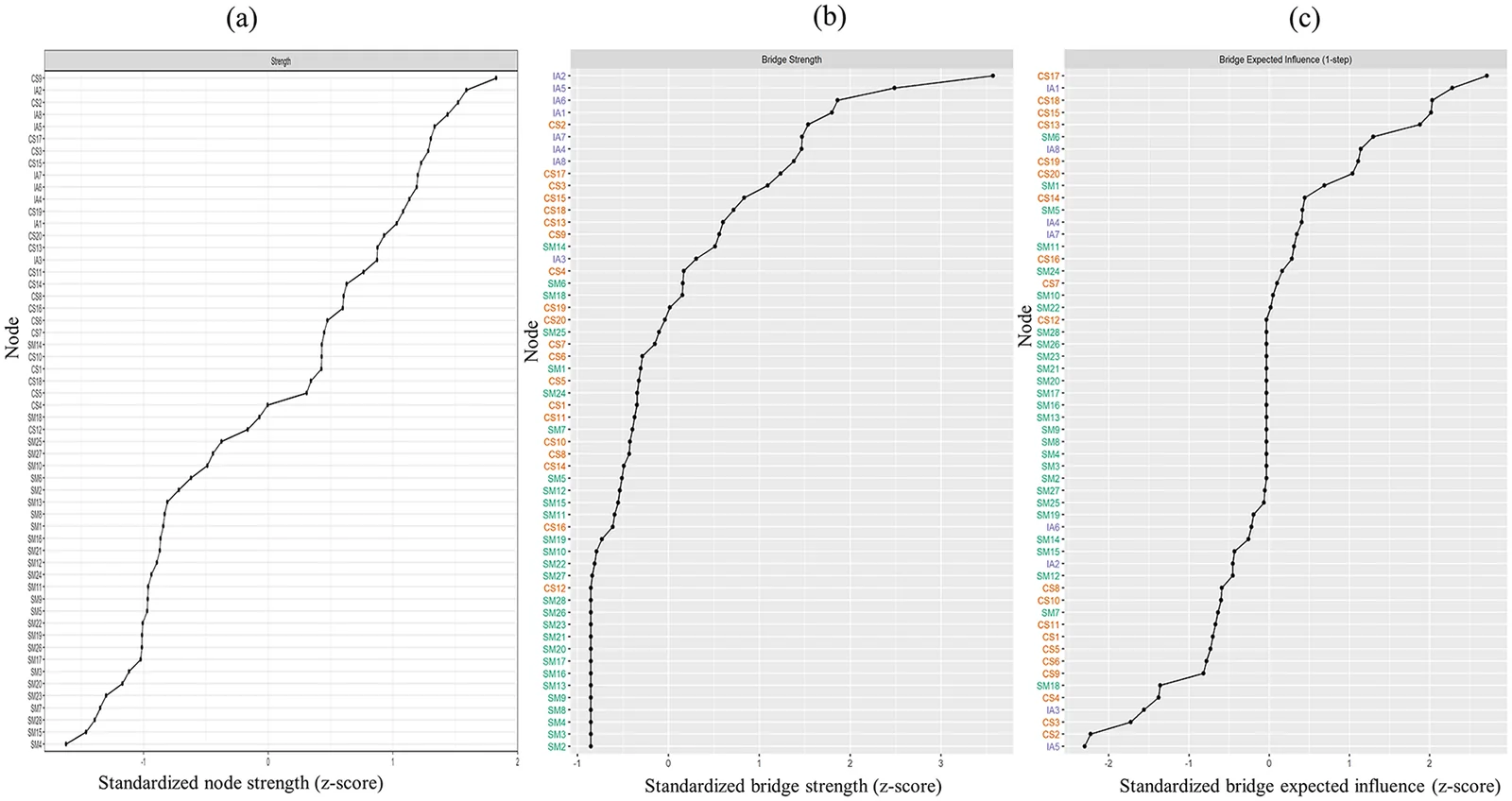
Node centrality and bridge centrality indices of the network. **(A)** Node strength; **(B)** bridge strength; **(C)** bridge expected influence (one-step).

#### 3.4.4 Network accuracy and stability assessment

To evaluate the accuracy and stability of the network estimates, 1,000 nonparametric Bootstrap samples were used to assess the accuracy of edge-weight estimates, and 1,000 case-dropping Bootstrap samples were used to evaluate the stability of node strength centrality. The results showed that the Bootstrap confidence intervals for stronger edges were generally relatively narrow, whereas those for some weaker edges were comparatively wider; therefore, weaker connections should be interpreted with caution. The case-dropping stability analysis showed that, as the proportion of the sample was progressively reduced, node strength estimates obtained from the reduced samples remained highly correlated with those derived from the original sample. The correlation stability coefficient (CS-coefficient) for node strength was 0.594. According to established recommendations, the CS-coefficient should not be lower than 0.25 and preferably should exceed 0.50 (Epskamp et al., 2018). Therefore, node strength centrality in the present study demonstrated good stability. Overall, the item-level network showed good edge-weight estimation accuracy and node strength stability, providing support for the further identification of important nodes within the overall network. The results of the network accuracy and stability analyses are presented in Figure 5.

**Figure 5:**
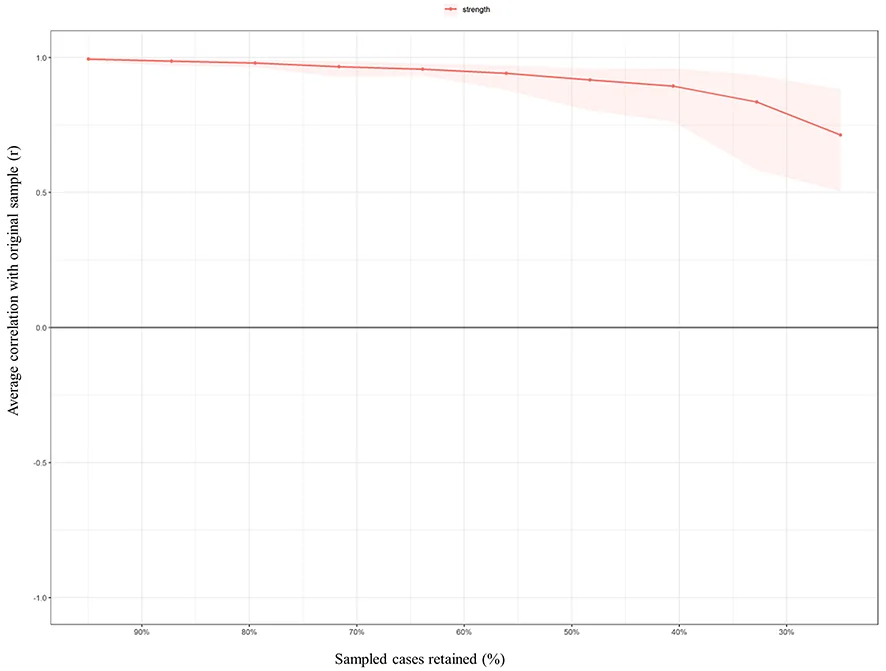
Network accuracy and stability estimates.

## 4 Discussion

### 4.1 Different types of sport motivation and PSNSU

The present study found that different types of sport motivation showed distinct patterns of association with PSNSU. Autonomous motivation was significantly and negatively associated with PSNSU, whereas controlled motivation and amotivation were significantly and positively associated with PSNSU. This finding is consistent with Self-Determination Theory, which proposes that different forms of motivational regulation have distinct functional meanings (Ryan and Deci, 2000). Autonomous motivation reflects a higher degree of self-determined regulation, with sport participation driven primarily by interest, value identification, and active choice; in contrast, controlled motivation is more strongly driven by external demands or internal pressures, whereas amotivation reflects a lack of clear behavioral intention and effective regulation. Compared with previous studies that have mainly examined the relationship in terms of overall sport motivation, physical activity levels, or general problematic Internet-related behaviors, the present study further distinguished among three types of sport motivation and found that they were associated with PSNSU in different directions. Previous research has generally suggested that higher-quality sport motivation or higher levels of physical activity are associated with lower levels of problematic Internet-related behaviors (Wu et al., 2026; Ren et al., 2026; Shan et al., 2025), whereas the present findings further indicate that this pattern does not apply uniformly across all forms of sport motivation: autonomous motivation was associated with lower levels of PSNSU, whereas controlled motivation and amotivation were associated with higher levels of PSNSU. This distinction suggests that the quality of motivational regulation underlying sport participation may provide more fine-grained information than overall motivation level or the amount of sport participation alone. Previous studies have also suggested that sport motivation may be associated with cognitive control, goal orientation, and broader processes of behavioral self-regulation (Liu and Li, 2023; Zhang et al., 2022). These psychological processes may provide a possible theoretical explanation for why different types of sport motivation show associations with PSNSU in different directions; however, these variables were not directly measured in the present study, and therefore it cannot be determined whether they constitute specific underlying mechanisms.

### 4.2 Indirect associations through positive coping and negative coping

The present study further found significant indirect associations between different types of sport motivation and PSNSU through positive coping and negative coping, with the indirect association of autonomous motivation being more strongly reflected in the positive coping pathway, whereas those of controlled motivation and amotivation were more strongly reflected in the negative coping pathway. This pattern of indirect associations is consistent with Self-Determination Theory, which proposes that different qualities of motivational regulation have distinct psychological functions, and the findings support Hypothesis 2 (Qiu et al., 2023; Du and Zhang, 2022; Peng et al., 2025). The results suggest that adolescents with higher levels of autonomous motivation may be more inclined to view difficulties in real life as manageable and adjustable. When facing stress, they may be more likely to maintain daily routines and respond through positive behavioral strategies, thereby reducing their reliance on online environments (Hu et al., 2025). In contrast, adolescents with higher levels of controlled motivation or amotivation may show less initiative when dealing with real-life problems. When confronted with academic pressure, interpersonal conflict, or emotional distress, they may be more likely to adopt avoidance or delayed responses. Under such circumstances, social networking use may gradually become a habitual means of relieving stress and diverting attention away from negative experiences (de Oliveira Barbosa et al., 2024; Günaydin et al., 2022). As this coping pattern becomes increasingly reinforced, online activities may shift from ordinary entertainment toward a form of psychological compensation, which may be associated with a higher risk of PSNSU. This finding is also consistent with the I-PACE model, which proposes that individual characteristics may be associated with problematic Internet-related behaviors through more proximal affective and cognitive processes (Brand et al., 2016, 2019); however, in the present study, the differences between sport motivation types and coping styles can be understood primarily in terms of the quality of motivational regulation emphasized by Self-Determination Theory. Positive coping may weaken the association between stressful experiences and PSNSU, whereas negative coping may amplify the influence of negative experiences on social networking use (Wu et al., 2024; Xu et al., 2024; Zhang et al., 2024). Therefore, the indirect associations through coping styles suggest that the statistical relationship between sport motivation and PSNSU is not limited to a direct association between sport participation and social networking use. This pattern is also consistent with broader psychological processes involving how adolescents interpret stress, select coping strategies, and regulate their own behavior (Howard et al., 2025; Luo et al., 2024; Wang et al., 2025). These findings suggest that the prevention of adolescent PSNSU should not focus solely on restricting social networking use, but should also place greater emphasis on jointly fostering sport motivation and adaptive coping styles to help adolescents develop more positive and stable ways of dealing with real-life problems.

### 4.3 Cross-sectional network analysis

The present study further used network analysis to reveal more fine-grained item-level associations among SM, CS, and PSNSU. Unlike mediation models, which mainly examine whether CS functions as an intermediate variable, network analysis focuses on specific psychological and behavioral components and reveals local connection patterns among SM, positive coping, negative coping, and PSNSU symptoms. The findings showed that the relationship between SM and PSNSU was not limited to a single linear association, but instead formed a complex network involving multiple coping-related nodes and PSNSU-related symptoms (Hirota et al., 2025; Wang et al., 2024). Centrality analysis showed that the positive coping node “change previous ways of doing things or one's own problems” occupied a central position in the network. This finding suggests that actively modifying one's behavior or problem-solving approach may represent an important psychological component linking adolescents' coping system with social networking use. Therefore, positive coping should not be understood merely as emotional comfort; it also reflects an individual's capacity to reorganize behavior and adjust problem-solving strategies under stressful conditions. When adolescents are able to actively modify maladaptive behavioral patterns, their reliance on social networking as a coping channel may be reduced (Castro et al., 2024; Tomaszek and Muchacka-Cymerman, 2021). Accordingly, this node showed relatively high statistical importance in the current cross-sectional network and may serve as a candidate factor for future longitudinal or experimental studies examining its relationship with changes in PSNSU. Among the PSNSU nodes, “thinking about logging onto social networking sites immediately after waking,” “failed attempts to reduce use,” “others considering the use excessive,” “using social networking when feeling depressed,” and “feeling anxious or restless when unable to access social networking sites” showed relatively high centrality. These findings suggest that adolescent PSNSU is not simply characterized by longer durations of social networking use. Rather, it may be more clearly reflected in the mutual reinforcement of urges to use, impaired control, emotional dependence, and withdrawal-like responses (Chen et al., 2024; Fournier et al., 2023; Peng and Liao, 2023). In particular, the urge to log on immediately after waking and failed attempts to reduce use may reflect habitual and automatic patterns of social networking use. Using social networking when experiencing low mood and feeling anxious when unable to access social networking sites further suggest that social networking use may gradually become a means of emotion regulation (Gioia et al., 2021; Iannattone et al., 2024). Therefore, interventions for PSNSU should not focus solely on reducing the duration of use, but should also address core symptoms such as urges to use, emotion-driven use, and impaired self-control. Bridge centrality analysis further revealed different functions of positive coping and negative coping within the network (Huang et al., 2022). Positive coping nodes mainly showed negative bridge effects. These nodes included talking about worries, viewing problems positively, rediscovering important aspects of life, changing previous ways of doing things, and maintaining one's own position. In the current network, these nodes mainly showed negative cross-construct connections with PSNSU-related nodes (Dong et al., 2024). In contrast, negative coping nodes mainly showed positive bridge effects. These nodes included relying on others to solve problems, passively waiting, temporarily avoiding problems, fantasizing that the situation would change, and self-comforting. In the current network, these coping nodes mainly showed positive cross-construct connections with PSNSU-related nodes (Ma et al., 2022). This pattern indicates that CS is not a homogeneous construct within the SM–CS–PSNSU network. Rather, it contains both potentially protective components and risk-related components. Positive coping may help interrupt the spread of PSNSU-related symptoms; positive coping nodes mainly showed negative cross-construct connections, whereas negative coping nodes mainly showed positive cross-construct connections, and this distinction warrants further examination in future longitudinal or dynamic network studies. Therefore, separating positive coping from negative coping provides a clearer understanding of how CS may connect SM and PSNSU. The present study makes several contributions. First, it incorporated SM, which is closely related to participation in physical activity, into research on PSNSU, extending previous studies that focused mainly on exercise behavior or physical activity levels. Second, the network analysis showed that the relationship between SM and PSNSU cannot be understood simply as a matter of “sport replacing social networking use”; rather, it involves interactions among multiple nodes related to positive coping, negative coping, and PSNSU symptoms. Third, the present study identified several key nodes, including active behavioral adjustment, emotional expression, positive cognitive appraisal, passive dependence, and avoidant waiting. These nodes may serve as candidate factors for further examination in future longitudinal or experimental studies. From the perspective of school physical education practice, rather than simply increasing students' sport motivation, greater attention should be paid to the quality of sport motivation. Teachers may foster more autonomous forms of sport motivation by using need-supportive teaching behaviors, such as providing appropriate choices, offering positive feedback, and creating supportive teacher–student interactions (Ahmadi et al., 2023). Future research should further examine whether changes in nodes such as positive coping, passive coping, avoidance, and dependent coping are associated with changes in PSNSU symptoms, and assess their potential intervention value on the basis of longitudinal or experimental evidence.

## 5 Limitations and future directions

Several limitations of this study should be acknowledged. First, the study employed a cross-sectional design. Although the findings revealed significant associations among SM, CS, and PSNSU, the causal direction and temporal ordering of these relationships cannot be determined. Significant indirect effects in cross-sectional mediation analysis only indicate that the data are consistent with the proposed statistical model, and alternative orderings of the variables may also be possible. Future studies should employ longitudinal designs, cross-lagged models, or intervention studies to further examine the long-term influence of SM on PSNSU and its potential psychological mechanisms. Second, the data were primarily collected through student self-report questionnaires and may therefore be affected by social desirability, recall bias, and common method factors. Future research could integrate multiple sources of data, such as teacher assessments, parent reports, objective physical activity records, and digital behavioral data on social networking use, to improve the reliability and ecological validity of the findings. In addition, this study used convenience sampling and collected data through an online survey, with participants drawn from adolescents in a specific region. Therefore, some degree of selection bias may exist, which may limit the representativeness and generalizability of the findings. Future studies could adopt more representative sampling strategies and include adolescents from a broader range of regions, school types, and population groups. Fourth, the present study focused primarily on sport motivation but did not directly measure adolescents' actual physical activity levels or sport participation behaviors. Therefore, although different types of sport motivation were significantly associated with PSNSU, the present study cannot determine whether higher levels of sport motivation translate into greater real-world sport participation, nor can it examine whether actual sport behavior is involved in the association between sport motivation and PSNSU. Future studies should simultaneously assess sport motivation and actual physical activity to further examine their relative roles in PSNSU and the relationship between them. In addition, although the present study combined mediation analysis and network analysis to examine the relationships among SM, CS, and PSNSU from both overall pathway and item-level perspectives, network analysis primarily reflects conditional associations among variables and cannot directly establish causal mechanisms. Future studies could employ longitudinal or dynamic network approaches to further explore changes in positive coping, negative coping, and core PSNSU symptoms over time. Moreover, future research could incorporate additional variables, such as self-control, loneliness, sleep quality, peer relationships, and family support, to develop a more comprehensive explanatory model.

## 6 Conclusion

The present study examined the relationships between different types of sport motivation and PSNSU among adolescents and further investigated the indirect associations through positive coping and negative coping, as well as the item-level network structure. The main conclusions are as follows. First, different types of sport motivation showed associations with PSNSU in different directions: autonomous motivation was significantly and negatively associated with PSNSU, whereas controlled motivation and amotivation were both significantly and positively associated with PSNSU. Second, significant indirect associations between all three types of sport motivation and PSNSU were observed through both positive coping and negative coping. The indirect association of autonomous motivation was more strongly reflected in the positive coping pathway, whereas those of controlled motivation and amotivation were more strongly reflected in the negative coping pathway. Third, network analysis further showed that positive coping and PSNSU were primarily connected through negative cross-community associations, whereas negative coping and PSNSU were primarily connected through positive cross-community associations. Several positive coping, negative coping, and PSNSU items also showed relatively high statistical importance within the network. Overall, by integrating construct-level and item-level perspectives, the present study revealed distinct patterns of association among different types of sport motivation, coping styles, and PSNSU, providing further evidence for understanding the psychological characteristics associated with adolescent PSNSU.

## Data Availability

The raw data supporting the conclusions of this article will be made available by the authors, without undue reservation.
